# Does resectoscope size play a role in formation of urethral stricture following transurethral prostate resection?

**DOI:** 10.1590/S1677-5538.IBJU.2014.0093

**Published:** 2015

**Authors:** Mustafa Günes, Muzaffer Oguz Keles, Cevdet Kaya, Orhan Koca, Zülfü Sertkaya, Mehmet Akyüz, Muammer Altok, Mehmet Umul, Muhammet Ihsan Karaman

**Affiliations:** 1 Department of Urology, Faculty of Medicine, Süleyman Demirel University, Isparta, Turkey; 2 Department of Urology Haydarpasa Training and Research Hospital, Istanbul, Turkey

**Keywords:** Urethral Stricture, Transurethral Resection of Prostate, Prostatic Hyperplasia

## Abstract

**Background and aims:**

To investigate the possible effect of resectoscope size on urethral stricture rate after monopolar TURP.

**Materials and Methods:**

A retrospective study of 71 men undergoing TURP was conducted at two centers’ from November 2009 to May 2013. The patients were divided into one of two groups according to the resectoscope diameter used for TURP. Resectoscope diameter was 24 F in group 1 (n=35) or 26 F in group 2 (n=36). Urethral catheter type, catheter removal time and energy type were kept constant for all patients. Urethral stricture formation in different localizations after TURP was compared between groups.

**Results:**

There was no significant difference between the two groups in terms of age, pre-operative prostate gland volume (PV), prostate-specific antigen (PSA), maximal urinary flow rates (Qmax), International Prostate Symptom Score (IPSS) and post-voiding residual urine volume (PVR). The resection time and weight of resected prostate tissue were similar for both groups (p>0.05). A statistically significant higher incidence of bulbar stricture was detected in group 2 compared to group1 (p=0.018).

**Conclusions:**

The use of small-diameter resectoscope shafts may cause a reduction in the incidence of uretral strictures in relation to urethral friction and mucosal damage.

## INTRODUCTION

Surgical treatment for lower urinary tract symptoms (LUTS) due to benign prostatic obstruction (BPO) is required in patients with certain indications or in the case of resistance to medical management (1). Transurethral resection of the prostate (TURP) still represents the gold standard in the operative management of benign prostatic hyperplasia (BPH) (2). However, TURP is recognized as the main cause of urethral strictures in patients older than 45 years old (3).

The incidence of urethral strictures after monopolar prostate resection varies between 2.2% and 9.8% (2). The main reason of urethral stricture after TURP is still unclear. On the other hand, it is well known that endoscopic instrumentation is one of the most common reasons for strictures of bulbar urethra. The improper urethral introduction of the resectescope, mucosal perforation related to the peno-scrotal angle and monopolar current leakage due to insufficient resectoscope isolation are the possible mechanisms for urethral stricture (2). Additionally, some other factors like the resectoscope size, the type and diameter of catheter, the duration of catheterization, resection time, patient age and urethral instrumentation have been investigated in relation to urethral stricture (4).

To the best of the authors’ knowledge, there is no wide literature exploring the effect of resectoscope size on formation of urethral stricture following TURP. In this study, we evaluated the effect of resectoscope diameter on urethral stricture after monopolar TURP.

## MATERIALS AND METHODS

### 

#### Patient data

After the approval of the local ethical committee, medical records of 71 men who underwent TURP were evaluated. The study was conducted in two centers. The patients were categorized in one of two groups according to the resectoscope size used for TURP. Resectoscope diameter was 24 (French) F in group 1 (n=35) or 26 F in group 2 (n=36).

A subject was eligible for enrollment in the study if the following criteria were met: at least 45 years of age and with a clinical diagnosis of BPH; prostate gland volume (PV) ≤ 80 mL; an International Prostate Symptom Score (IPSS) ≥20; Quality of Life (QoL) ≥3; maximal urinary flow rates (Qmax) below 15 mL/s; failed conservative medical therapy and thus surgical treatment indication for TURP; normal urinary bladder function.

Post-voiding residual urine volume >400 mL, indwelling urethral catheter prior to surgery, history of prostate surgery, urethral stricture, bladder stone, neurogenic bladder dysfunction and presence or suspicion of prostate malignancy were the main exclusion criteria of the study. In addition, specific perioperative complications including urinary retention and symptomatic culture confirmed bacterial urinary tract infection and patients with prolonged catheterization time due to hematuria and operation time >90 minutes were additional exclusion criteria for the study. Also we have excluded the patients with a diagnosis of meatal stricture prior to TURP.

#### Clinical Evaluation

All patients underwent a general and urological standard evaluation before surgery, including physical examination with digital rectal examination, urine analysis, transrectal ultrasound (TRUS) volume measurement of the prostate, blood sample analysis including prostate-specific antigen (PSA) level, Qmax, postvoid residual urine volume assessment (PVR), QoL assessment and self assessment by IPSS. Both groups have been compared according to PV, PSA, resection time, IPSS and urethral stricture required intervention.

### Surgical technique

All of the operations were performed by two expert surgeons with over 10 years experience. TURP was performed using a standard technique; 26F continuous flow resectoscope (Karl Storz GmbH & Co. KG, Tuttlingen, Germany) with mono-polar loop electrode was used for first group and 24F single flow resectoscope (Karl Storz GmbH & Co. KG, Tuttlingen, Germany) with mono-polar loop electrode was used for second group. TURP was performed with the electrocautery system (Valleylab Force FXTM, Boulder, CO, USA); the settings for cutting and coagulation were 140 (Watt) W and 80 W, respectively. Resection was performed using both resectoscopes; Resectisol® (Eczacibasi-Baxter, Istanbul, Turkey) solution was used as irrigation fluid during surgery. Postoperatively 22F three-way latex Foley catheter was used in all patients and catheters were taken out 72 h after the operation.

#### Patients follow-up

The patients were reassessed at 3-month intervals in the outpatient clinic. Urinalysis, uroflowmetry, and ultrasonographic measurement of postvoid residual urine volume were performed. In patients with a typical flow pattern of a stricture or peak flow less than 15 mL/s, a retrograde uretrogram was performed to exclude urethral stricture. Furthermore, cystoscopy and internal urethrotomy was performed in the presence of urethral strictures.

### Statistical analysis

Statistical analysis was performed using the SPSS 15.0 software package (SPSS, Inc., Chicago, IL). The Kolmogorov-Smirnov normality tests were used to determine if the data set was well-modeled by a normal distribution. t test was used for comparison of groups for examined variables and results were given as mean ± standard deviation. Two proportions Z test were used for bulbar and meatal proportions. A p-value of 0.05 or less was considered statistically significant.

## RESULTS

A total of 71 patients were included in the study. Patient related, operational and hospitalization characteristics of both groups are reported in Table-1. There was no significant difference between the two groups in terms of age, PV, PSA, Qmax, IPSS and PVR. The mean age was 64.2±8.6 (49-83) years old for group 1 and 67.3±8.1 (48-85) years old for group 2. Mean prostate volume was determined as 51.6±15.5 mL (24-80) mL in group 1 and 51.9±13.0 (30-80) mL in group 2. The mean operative time was 54.8±18.5 (25-90) minutes in group 1 and 56.19±16.9 (30-90) minutes in group 2.

**Table 1:** Preoperative baseline patients characteristics.

Parameter	24F (n=35)	26F (n=36)	p value
Age(years old)	64.2±8.6	67.3±8.1	0.130
PSA(ng/mL)	4.1±4.6	4.6±4.5	0.581
PV(mL)	51.6±15.5	51.9±13.0	0.907
IPSS	22.9±2.2	23.4±2.0	0.363
QoL	5.0±0.6	5.1±0.6	0.108
PVR (mL)	96.3±50.7	92.8±49.1	0.407
Qmax (mL/s)	8.6±1.8	9.1±1.9	0.335

Values are given as mean ± standard deviation.

**PSA** = prostate-specific antigen; **PV** = prostate gland volume; **IPSS** = International Prostate Symptom Score; **QoL** = quality of life; **PVR** = postvoid residual urine volume; **Qmax** = maximum flow rate.

All patients had at least 1 year of follow-up. The improvements in the Qmax and PVR are illustrated in Table-2, and were comparable between the two groups with no significant differences between them. The resection time and weight of resected prostate tissue were similar for both groups (Table-2). All cases had histopathology reported as BPH.

**Table 2:** Operational data and postoperative findings.

Parameter		24F (n=35)	26F (n=36)	p value
Resection time (min)		54.8±18.5	56.2±16.9	0.747
Resected prostate tissue(g)		26.5±6.9	27.3±7.0	0.645
**Postoperative Qmax (mL/s)**				
	1 month	19.1±4.6	16.9±5.9	0.087
	3 month	18.1±5.8	17.7±5.6	0.920
	12 month	19.3±5.2	18.8±5.1	0.940
**Postoperative PVR (mL)**				
	1 month	45.7±15.9	45.0±16.2	0.250
	3 month	38.9±10.5	37.9±12.2	0.401
	12 month	32.0±14.2	30.7±13.5	0.230

Values are given as mean ± standard deviation.

**Qmax** = maximum flow rate; **PVR** = postvoid residual urine volume

Urethral strictures were detected after six months postoperatively. The patients with urethral stricture following TURP in group 2 had a significantly lower Qmax compared with group 1 (Table-3).

**Table 3:** Qmax and PVR values of patients with uretral stricture following TURP.

Parameter	Urethral strictures		p value
	**24F (n=3)**	**26F (n=5)**	
Qmax (mL/s)	11.6±3.1	5.4±1.1	0.041
PVR (mL)	139.2±53.2	149.4±61.3	0.620

Values are given as mean ± standard deviation.

**Qmax** = maximum flow rate; **PVR** = postvoid residual urine volume.

Bulbar urethra and meatus strictures were compared for both groups (Table-4). We have not detected a significant difference among groups in terms of meatal stricture. A statistically significant difference was determined among groups in terms of bulbar stricture formation (p=0.018). All of these patients needed a second intervention. Reoperations consisted of visual urethrotomy in five patients and external meatotomy in three patients. Repeated dilatation was performed in two patients. In group 2, one patient required further surgery for urethral stricture (perineal urethroplasty). Temporary stress incontinence did not occur in any patient postoperatively.

**Table 4:** Incidence of urethral stricture in the study groups.

Variables	N(%)	N(%)	p value*
	**24F**	**26F**	
Bulbar urethra	1 (2.9%)	4 (11.4%)	0.018
Meatal urethra	2 (5.7%)	1 (2.9%)	0.386
Total	3 (8.6%)	5 (%13.9)	0.619

* Z test.

## DISCUSSION

TURP is still considered the surgical gold standard for the management of symptomatic BPO in prostates between 30 and 80 cc (5). Urethral strictures in different locations are mentioned as the major late complication of monopolar TURP (6). The type of the urethral catheter, length of catheterization period, diameter of the resectoscope, duration of resection and patient related factors such as age have been considered as risk factors for the development of urethral stricture after TURP (4,7,8). All of these variables were kept constant, in order to investigate the possible effect of changes on resectoscope diameter, for all patients. In our study, the incidence of urethral stricture was determined as 8.6% and 13.9% for groups 1 and 2, respectively. These results are compatible with randomized controlled clinical studies (0.0% – 14.7%) (6). There was no significant difference among groups in terms of meatal stricture. The underlying reason for the slightly increase in meatal stricture incidence in group 1 may be related to the use of non-continuous resectoscope shaft. Performing TURP with such type of resectoscope may cause increased meatal stricture incidence in relation with the reciprocation of the shaft in the axial axis during bladder decompression.

Although the decrease in the incidence of urethral strictures is an expected result of technological development, the diameter of instruments remains an important factor for urethral stricture formation. Despite the introduction of new technologies such as holmium laser, green light laser and bipolar TUR in daily urological practice, the incidence of urethral stricture after TUR has not changed significantly (6,9–12). Furthermore, it has been observed that a higher incidence of urethral stricture may occur following these new techniques in comparison with monopolar TURP (13–17). The usage of broader resectoscope shafts is the common feature of the studies reporting higher urethral stricture incidence (11,15,16).

The clinical results of plasmakinetic resection of the prostate with 27 F resectoscope and monopolar TURP with 26 F resectoscope have been compared by Erturhan et al. Urethral injury and meatal strictures were detected more often in 27 F group. These injuries have been correlated with partial rupture of bulbo-membranous urethra during first entrance (16).

In a prospective, randomized trial, Tefekli et al. compared 27 F bipolar and 26 F monopolar TURP and reported a statistically significant higher urethral stricture rate in 27 F group. The authors suggested that the increase may be related with high-ablative energy use as well as large resectoscope sheath use (15). In a prospective, non-randomized study, Chen et al. have compared laser vaporization and conventional TURP in terms of development of urethral stricture and found significantly lower urethral stricture incidence in laser vaporization group. However, the higher urethral stricture incidence in TURP group was correlated with large-diameter shaft use (23F vs. 26F) (11).

The studies indicating the increase of urethral stricture rate with the use of broader resectoscopes are not standardized in terms of the use of different energy technologies and urethral catheter removal time. In our study, factors related with development of urethral stricture such as the energy type, urethral catheter type and length of catheterization period were standardized.

In our study, bulbar stricture was significantly higher in 26 F group (p=0.018). The detection of bulbar strictures in penoscrotal angulation zone, in both groups, supports the mucosal damage caused by the friction of the resectoscope shaft and scar tissue formation due to submucosal fluid leakage hypothesis (3).

The higher incidence of bulbar stricture in 26 F group indicates the importance of resectoscope diameter. However, there is still a need for large scaled, prospective, randomized clinical studies with standard energy type and different resectoscope sizes.

Our limitations for current study include the retrospective nature and relatively small sample size for study groups, the use of continuous–flow resectoscope in 26 F group and noncontinuous-flow resectoscope in 24 F group.

In conclusion, the use of small-diameter resectoscope shafts may cause a reduction in the incidence of uretral strictures in relation to urethral friction and mucosal damage. Thus, we can cope better with this late complication of TURP. We recommend the use of small-diameter resectoscope shaft during endo-urological interventions such as TURP.
